# The Link between Inequality and Population Health in Low and Middle Income Countries: Policy Myth or Social Reality?

**DOI:** 10.1371/journal.pone.0115109

**Published:** 2014-12-11

**Authors:** Ioana van Deurzen, Wim van Oorschot, Erik van Ingen

**Affiliations:** 1 Sociology Department, Tilburg University, Tilburg, The Netherlands; 2 Department of Sociology, Leuven University, Leuven, Belgium; The George Institute for Global Health, India

## Abstract

An influential policy idea states that reducing inequality is beneficial for improving health in the low and middle income countries (LMICs). Our study provides an empirical test of this idea: we utilized data collected by the Demographic and Health Surveys between 2000 and 2011 in as much as 52 LMICs, and we examined the relationship between household wealth inequality and two health outcomes: anemia status (of the children and their mothers) and the women' experience of child mortality. Based on multi-level analyses, we found that higher levels of household wealth inequality related to worse health, but this effect was strongly reduced when we took into account the level of individuals' wealth. However, even after accounting for the differences between individuals in terms of household wealth and other characteristics, in those LMICs with higher household wealth inequality more women experienced child mortality and more children were tested with anemia. This effect was partially mediated by the country's level and coverage of the health services and infrastructure. Furthermore, we found higher inequality to be related to a larger health gap between the poor and the rich in only one of the three examined samples. We conclude that an effective way to improve the health in the LMICs is to increase the wealth among the poor, which in turn also would lead to lower overall inequality and potential investments in public health infrastructure and services.

## Introduction

During recent years, income inequality has been flagged as a true villain of our times, a root cause of a wide range of social problems. Especially the relationship between inequality and health was extensively discussed among scholars and public policy practitioners [1]–[5]. Organizations such as United Nations (UN), Save the Children and the World Health Organization (WHO) stressed that, in order to improve health, tackling inequalities is a priority that needs to accompany the efforts to alleviate absolute poverty in the low and middle income countries (LMICs) [6]–[8]. For example, the UN Task Team's work on the post-2015 UN Development agenda made this point very clear: “High levels of inequalities can jeopardize the well-being of large segments of the population […] and have subsequent effects on health, nutrition and child development” ([7]:6). Furthermore, WHO emphasized that inequality is not only relevant for improving the average health but also for closing the health gap between the rich and the poor in the LMICs: “In any country, economic inequality… needs to be addressed to make progress towards health equity.” ([6]:120).

Although the LMICs are the focus of these recommendations, evidence was often cited from studies that examined samples of high income countries (HICs) (e.g., [9]). The evidence for a detrimental effect of income inequality on health among wealthy countries is extensive - by now more than 160 studies looked at this relationship [10]. Furthermore, a recent meta-analysis of multi-level studies conducted in HICs found a small but significant negative effect of income inequality on individuals' health [11]. The authors cautiously concluded: “if the inequality-mortality relation is truly causal, then the population attributable fraction suggests that upwards of 1.5 million deaths (9.6% of total adult mortality in the 15–60 age group) could be averted in 30 OECD countries by levelling the Gini coefficient below the threshold value of 0.3” ([11]:7). However, in the light of the profound cultural, economic, and political differences between the LMICs and the HICs, it is questionable whether such findings from the HICs can be transferred to fundament policies targeted at improving population health in the LMICs.

We note that the number of studies that examined the inequality-health nexus among the LMICs is far more limited than those that focused on HICs. For instance Biggs, King et al. (2010), found an unexpected result in a sample of Latin American countries, i.e., the increase in inequality measured by Gini Index of income was associated with a significant increase in life expectancy and with a significant decrease in mortality and infant mortality rate. Pop, van Ingen et al. [12] corroborated these conclusions: in their ecological analysis of time series data the authors found that increasing inequality in incomes was related to increasing life expectancy in the low income countries but not in the middle and high income ones. Other studies used mixed samples, pooling together low, middle and high income countries, e.g., Babones [13] who found a significant negative relationship between income inequality and life expectancy and infant mortality. However, a different study that examined a mixed sample of countries found that the negative relationship between inequality and life expectancy or infant mortality rate was not robust when controlling for unmeasured heterogeneity [14].

In relation to specific health measures such as child mortality rate, which is an important target of the Millennium Development Goals [15], an ecological study among 46 developing countries found income inequality not to be associated with under 5 mortality rate [16], while another ecological study found higher levels of income inequality to relate to higher levels of infant mortality rate [17]. Rajan, Kennedy and King [18] found that income inequality in India was not associated to child mortality rate when it was measured at state level but the relation was positive for the district level analysis. Another study that estimated the relationship between inequality and child mortality rate at the level of neighborhoods in Rio de Janeiro, Brazil, found a statistically not significant relationship [19].

The brief overview of the literature that examined the relationship between inequality and health in LMICs did not aim to be comprehensive but we wished to point out the contradictory results from previous studies. It is clear that there is still much work to be done in order to better understand why these inconsistencies have emerged. This is also our main goal in the present study: we aim to expand and contribute to the literature by examining the relationship between inequality and health and health inequality among the LMICs, with a focus on disentangling potential mechanisms at work. In order to address this aim we utilized measures of health with high cross-country equivalence and we employed multilevel models that allow separating compositional from genuine contextual effects [20].

**Figure 1:**
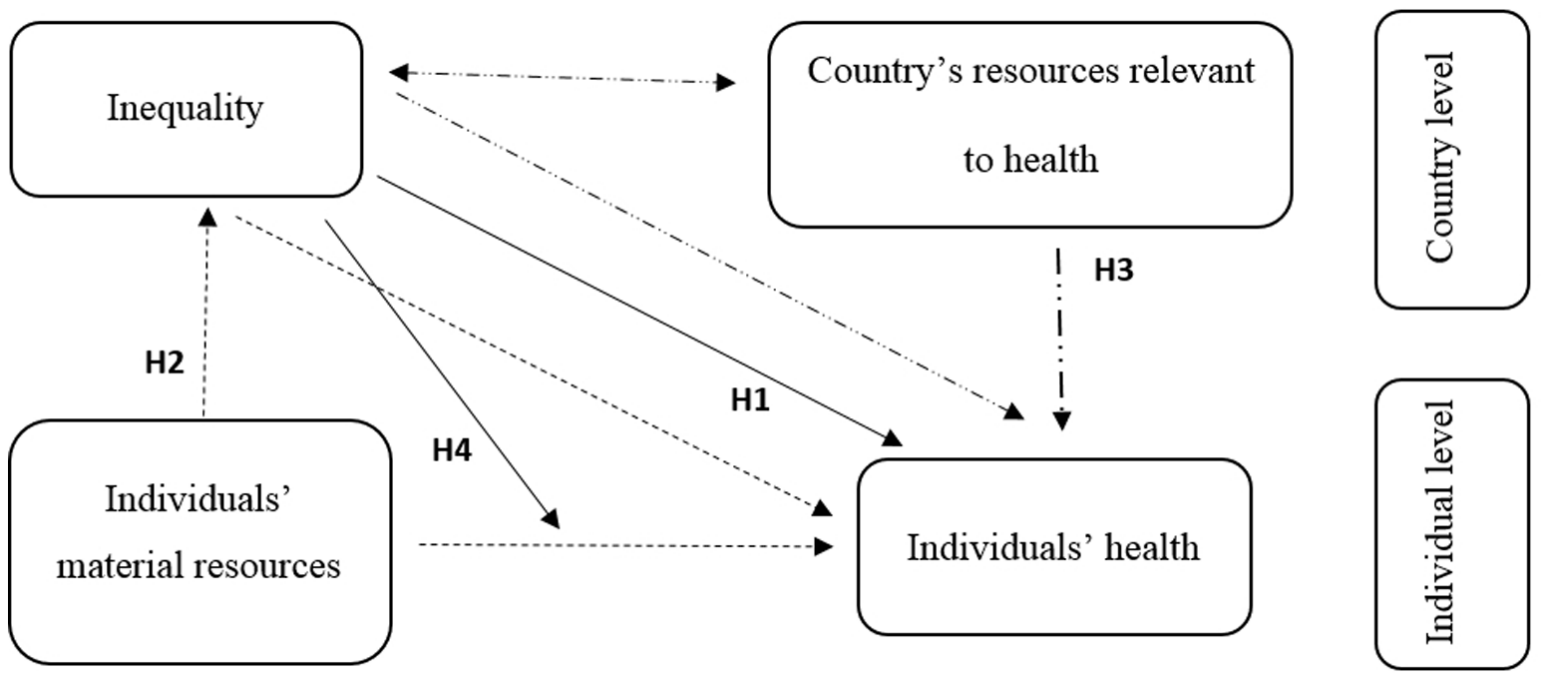
Graphic representation of the hypotheses in our study. *Fig. 1 notes*: Mediation paths depicted using different line types.

In the next sections we examine two potential explanations of the relationship between inequality and health: (1) the position stating that any relationship between inequality and health is just a statistical artefact due to composition effects, and (2) the position stating that inequality relates to population health via its relationship with the level and coverage of those country level resources that are relevant for improving health, i.e., the health services and infrastructure. The research questions that guide our study are: (1) to what extent is inequality associated with the health of individuals living in LMICs?; (2) is there evidence for a genuine contextual effect of inequality on health, independent of composition effects due to the population's structure?; (3) to what extent is a potential contextual effect of inequality on health mediated by the country's resources relevant to health? We discuss below the two potential explanations of the relationship between inequality and health and derive corresponding hypotheses that are presented in a graphic form in Fig. 1.

### The statistical artefact argument

Gravelle [21] argued that the relationship between inequality and health is a statistical artefact due to the non-linear relationship between material resources (income, wealth) and health at individual level. Take as an example of this argument a society that undergoes a process of redistribution of wealth from the rich to the poor. The consequence is that the living circumstances of the poor will improve with beneficial consequences for their health. However, the health gains among the poor are larger than the health loss of the rich as consequence of the wealth redistribution. Also, as a result of redistribution, the inequality will decrease. Thus, between two societies with the same level of overall wealth, the one with lower inequality will have better aggregated population health.

Research among HICs concluded that, at least in this context, the relationship between inequality and health is not entirely compositional [13], [22]. However, this type of reasoning could be particularly pertinent for the LMICs because of the lack of extensive welfare arrangements that would counteract the effects of poverty. Official figures estimate that more than 40% of the African population cannot secure enough food on a day to day basis [23], thus the level of absolute poverty is extremely high. Next, in the LMICs the access to the health-related goods and services is strongly related to the level of individual resources because of the high share of the private financing of the health sector, either via private insurance or out-of-pocket payments [24]–[26]. In addition, most LMICs do not have special programs to protect the poor or allow them a fair access to health services. It is thus clear that in the LMICs bad material circumstances can have disastrous consequences for individuals' health. Consequently, the level and distribution of material resources in the population could (at least partially) explain an observed relationship between inequality and the average societal health.

### Inequality and the countries' resources relevant to health

The “statistical artefact” proposition does not exclude the possibility that inequality might also have a genuine contextual effect on health. In this contribution, we explore one such possible mechanism – via the level of those country's resources that are most relevant to health, i.e., the health infrastructure and services available for the population.

Economists have argued that inequality has short and long run consequences for the organization and development of societies [27], resulting in a strong negative relationship between inequality and investments in public goods such as the health services and infrastructure. Several explanations for this theoretical and empirical observation were advanced. First, a self-interest mechanism might be at work: in countries with high inequality, the small rich elite is not eager to offer the resources needed for the poor majority to elevate and ultimately challenge its position [28]. For instance, the small rich elite would not be motivated to facilitate the access to health services by reducing the level of out-of-pocket payments. Second, environments with higher inequality have high levels of socio-political instability, which in turn will most likely also facilitate a self-interest attitude [29]. This mechanism could be exemplified by the observation that the majority of the health expenditures in the LMICs are directed toward hospitals located in cities, where the rich population resides, while the medical posts in rural areas are severely under-funded [24]. Third, economic literature has shown that inequality leads to underdevelopment and reduced growth [30], [31], resulting in low levels of resources available for investments in public goods, even if the political will to do so would exist. Last but not least, Lynch [32] argued that inequality is a result of a specific historical, political and economic development that also shaped a particular country's infrastructure through specific policies and arrangements affecting education, health, labor market, etc. The arguments of this author imply that inequality has a spurious relationship with health because it reflects effects of unmeasured characteristics of the country's infrastructure. In contrast, the first three mechanisms from the economic literature argue for a causal relationship between inequality and the country's resources that are relevant to health.

Summing up the above theoretical arguments on the two potential mechanisms linking inequality to health, we expect that in LMICs with higher inequality the average health to be worse than in LMICs where the inequality is lower (*H1*). Next, we expect that the strength of this relationship to become weaker when the material circumstances of individuals (*H2*) and the country's resources relevant to health (*H3*) are taken into account.

The above arguments focused only on the relationship between inequality and the average level of health. However, it is also possible that inequality has a different relationship with the health of the poor and of the rich. If we accept the idea that inequality deters the development and investments in public health services and infrastructure, one can argue that rich individuals have the advantage of more resources that can protect them from low quality public health services, e.g., they can access private clinics or seeks medical help outside the borders. Poor individuals, deprived of material possibilities, have to use what is available to them, and this might contribute to widening the health gap. Thus, in LMICs with higher inequality, the health gap between the rich and the poor is expected to be higher, in other words, the health inequality to be higher (*H4*).

## Data and Methods

### Selection of the data

We utilized individual level data collected by the Demographic and Health Surveys (DHSs) project, funded by the United States Agency for International Development [33]. The specific surveys used are nationally-representative household surveys that provide data for a wide range of monitoring and impact evaluation indicators in the areas of population, health, and nutrition. Data collection is typically conducted every 5 years using instruments with similar questions, although the sample is not identical from wave to wave. The data used in this study was collected among all eligible women in the selected households, i.e., women of reproductive age, usually between 15 and 49 years old, and their children with the age lower than 71 months.

Selection of the countries and years in our analyses was dictated mainly by the choice of dependent variables – the particular information is not collected in every country, every wave or for all the surveyed population. We limited the time span of our data to the period around 2000 to 2011 in order to ensure that enough contextual information is available. Because we are interested in differences between countries, we decided to pool together the different waves of data collected in one country. Next, we collected contextual data as average across the years between waves, or, when only one wave was available, as average for five years prior and including the year of data collection. Unfortunately we had to eliminate countries where the contextual data was not available (e.g., Zimbabwe).

### Dependent variables

We focus on two measures of health: anemia status of women and of their children and the women's experience of child mortality, i.e., the death of at least a child born in the last 5 years. Our choice of health measures was guided by several criteria. First, the health measures had to comply with the assumptions of comparative research, i.e., they had to be equivalent between countries. The anemia status was assessed by collecting blood samples in the field, that were afterward analyzed in specialized labs [34]. The advantage of this method is the use of standard medical tests and cut points, that ensures a higher degree of cross-country measurement equivalence. The mothers' experience with child mortality was calculated from their detailed birth history covering 5 years prior to the date of interview. We do not have reasons to suspect that the interviewed women lied or were unaware about their births.

Second, the health measures had to be relevant health concerns. On the one hand, reducing child mortality rate is one of the most important priorities of the Millennium Development Goals [15]. Furthermore, child mortality is a widely accepted population health indicator when examining the inequality-health relationship [5], [14], [35], [36]. Our variable is the translation of the ecological measure of under 5 mortality rate from country level to individual level. On the other hand, anemia is particularly relevant when examining women's health, i.e., it was linked to more frequent hospitalization and is considered an indirect cause of maternal mortality [37]–[40]. In relation to children's health, anemia was linked to poor cognition and motor development, but also to education achievement and behavioral problems [41]. These pieces of evidence position anemia as a public health problem.

Third, these health measures have a strong relationship with the material circumstances of individuals (e.g., availability of good quality nutrition, shelter and overall living conditions) [42], but child mortality is also dependent on the availability of medical assistance and infrastructure [16], [43]. We believe that the different relationship between our two health measures and individual and country specific circumstances will help shed more light on the mechanisms linking inequality to health in the LMICs and improve the robustness of our conclusions.

Fourth, from a practical point of view enough data had to be available in order to allow estimation of multilevel models.

*Anemia* is a condition in which the blood has a lower than normal number of red blood cells, or when the red blood cells do not contain enough hemoglobin, a protein-based component that helps cells carry oxygen throughout the body. Anemia was diagnosed with a blood test [34]. The anemia status was re-coded by the DHSs team into 4 categories, ranging from “severe”, “medium”, and “mild” to “no anemia”. For reasons of parsimony, we recoded this variable into 2 categories: “no anemia” vs. “any sign of anemia”. In our models “no anemia” was the reference category. Standard DHS protocol requires that informed consent be obtained from participants in anemia testing and that confidentiality be ensured. After receiving the authorization to download the biomarker information, the data was treated as confidential, and no effort was made to identify any household or individual respondent interviewed in the survey.

*The experience of child mortality* was calculated using birth history information. We determined for each woman if any of her children born maximum 5 years before the survey died and contrasted this category to women who did not experience the death of a child born during the same time interval (the reference category).

### Individual level variables

#### Material resources of the individuals

We measured the material resources of the individuals by means of an asset-based household wealth index. The DHSs do not provide a measure of individual income because of the difficulties associated to the collection of this information in the LMICs, e.g., the extension of informal labor agreements or the significant size of population that subsists on agriculture. Thus, in the LMICs, providing a correct estimation of household income is subject to serious bias but respondents can answer accurately to questions about their assets.

The lack of income information in the DHSs does not create an impediment for our analysis. The costs of illness in the LMICs are frequently above 10% of household income [26]. When faced with the costs of illness, households have specific coping strategies among which the most important are converting assets into currency or reducing their consumption. It is thus clear that in the LMICs assets are a more important resource for health than income. In addition, it is generally accepted that assets are good indicators of the long term socio-economic position in the LMICs.

The asset-based wealth index was calculated using easy-to-collect data on a household's ownership of selected assets, such as televisions and bicycles; materials used for housing construction; and types of water access and sanitation facilities. DHSs collected data on a number of identical assets in all countries and, in order to accommodate geographical and economic differences, e.g., owning a boat is not a relevant asset in a desert country, also collected data on several country specific assets. Subsequently, the assets based indexes are valid indicators of the wealth differences within a country in a specific time point, but they cannot be directly compared between countries and time points. The cross-country comparative research needs an index that uses the same criteria for rating households between countries and years [44].

In order to develop a measure that is comparable between countries and years we followed the method proposed by Smits and Steendijk [45] to derive the International Wealth Index (IWI). IWI ranges from 0 to 100 and it had a mean of 42.3 (SD: 28.6) in the women anemia sample, 33.8 (SD: 27.3) in the children anemia sample and 35.2 (SD: 27.9) in the child mortality sample. In our analyses we used dummies based on quintiles calculated for each of the three sample, with the poorest 20% as reference category. Note that this method of calculating the wealth quintiles resulted in an absolute wealth hierarchy (across all countries in a sample) and not a relative one (within each country).

### Contextual measures

#### Inequality

Because of the difficulties of collecting reliable income information in the LMICs, asset-based wealth indexes are regarded to be a more accurate estimation of the real economic conditions of the household. This is the reason why recent studies that explored effects of inequality on health among LMICs used inequality measures based on possession of assets [46], [47], a course of action that we also followed. *The household wealth inequality* was based on the IWI measure that we derived at household level, which we used to calculate a Gini Index of Household Wealth.

#### The country's resources relevant to health

From the World Health Organization statistics website we first collected three measures: a measure of *governmental spending on heath* measured in PPP international $; a measure of *private financing of health* as the percentage of total health spending financed by private insurance and out-of-pocket payments; and a measure of the *coverage of basic health services* as the percentage of children under 1 year that received measles vaccine, measure that is also considered a good estimation of the effectiveness of the health sector in the LMICs [24]. In addition to these contextual measures we also used as a contextual control variable the *level of wealth of the country* measured as GDP per capita PPP constant 2005 international dollars derived from the World Development Indicators dataset 2012 [48].

### Control variables

We also included in our analyses a set of individual level control variables that might confound the relationship between health and material resources. We first used a set of control variables that were common for both dependent variables. *Education level* (of the mother) was provided as a categorical variable that differentiated between women with no education (reference category), primary education, secondary education and tertiary education. *The age* of the women (mothers) was used in the analyses as dummies of 5 years intervals with the age 15 to 19 years old as reference category. *Residence*: a variable recording whether the respondent lived in urban or rural area. *Marital status* of the women (mothers) was measured as categorical variable with 3 categories: those women never married (reference category), those that at the time of the interview were married/living together and those that used to be in a relationship (widows, divorced or not living together with the partner for other reasons). The variable *number of household members* measured the size of the household.

The specific control variables are the following: 1) for the dependent variable anemia status, women sample, we included *pregnancy status* and whether the woman was *breastfeeding* or not, because these two situations have a significant effect on the chance to be tested with anemia [49]; 2) for the dependent variable anemia status, children sample, we included the *anemia status of the mother* because previous research has shown that infants born to anemic mothers have higher chances to be also tested with iron deficiency [50]; 3) for the dependent variable experience of child mortality, we included the *number of children born during the last 5 years*, as the chance to experience child mortality is higher with more children born.

Our working data samples consists of: (1) a sample of 373735 women nested in 33 countries for whom we have information on anemia status; (2) a sample of 152485 children with age less than 71 months nested in 30 countries, for whom we have information on their anemia status and (3) a sample of 455692 women nested in 52 countries for whom we have information on the experience of child mortality. A summary of the countries and waves in the analyses is included as supplementary material. We present descriptive information on the variables in our models in Table 1.

**Table 1:** Descriptive statistics.

	Women anemia sample a		Children anemia sample b		Experience of child mortality c	
	Mean/Proportion	Min/Max	Mean/Proportion	Min/Max	Mean/Proportion	Min/Max
Anemia status (yes = 1)	40.2%	0/1	58.7%	0/1		
Experience of child mortality (yes = 1)					8.6%	0/1
Household wealth	42.3 (28.6)	0/100	33.8 (27.3)	0/100	35.2 (27.9)	0/100
Primary education (of the mother)	27.5%	0/1	32.7%	0/1	33.7%	0/1
Secondary education (of the mother)	37%	0/1	28.2%	0/1	27.1%	0/1
Tertiary education (of the mother)	10.2%	0/1	6.8%	0/1	7.1%	0/1
Age 20–24	17.6%	0/1	24.5%	0/1	24.6%	0/1
Age 25–29	16.2%	0/1	29.3%	0/1	27%	0/1
Age 30–34	13.9%	0/1	20.7%	0/1	19.4%	0/1
Age 35–39	12.7%	0/1	12.7%	0/1	12.9%	0/1
Age 40–44	11%	0/1	6%	0/1	6.4%	0/1
Age 45–49	9%	0/1	1.8%	0/1	2.2%	0/1
Residence: rural	55.5%	0/1	64.8%	0/1	63.1%	0/1
Marital status: married	67.7%	0/1	92.2%	0/1	88.8%	0/1
Marital status: was married	7.4%	0/1	5.2%	0/1	7.1%	0/1
Number household members	6 (3.3)	1/74	6.7 (3.5)	2/74	6.6 (3.6)	1/74
Breastfeeding	20.5%	0/1				
Pregnant	6.4%	0/1				
Mother is anemic			42.9%	0/1	0/1	
No. children born last 5 years					1.4 (.6)	1/8
Gini Index of household wealth	34.9 (12.3)	7/53	35 (12.5)	7/53	33.1 (12.6)	7/53
Private financing of health	58.3 (15.3)	34.6/86.3	57.8 (15.2)	34.6/86.3	55.8 (15.6)	28.2/86.3
Measles vaccination	78.8 (14.8)	48.2/97.4	79.2 (14.9)	48.2/97.4	81.9 (13.8)	36.8/97.8
GDP per capita	2091.3 (1796.6)	275/7510.3	2100.9 (1822.5)	275/7510.3	2800.8 (2820.8)	130.2/14603.1

*Note*: continuous variables in their original metric, before transformations. SD in parenthesis when applicable.

a : women anemia sample, 373735 women in 33 countries.

b : children anemia sample, 152485 children with age less than 71 months in 30 countries.

c : experience of child mortality sample, 455692 women in 52 countries.

### Method of analysis

In order to formally test our hypotheses, we estimated binary logistic multilevel models, separate for each sample. We eliminated the missing values on the dependent and independent variables. The level of missing values for the dependent variables was low, e.g., only 8.7 percent of the women selected for anemia tests did not have a valid measurement. The level of missing values for the independent variables was also very low. We standardized the independent continuous measures at contextual or individual level (Mean = 0, SD = 1). To account for possible period effects, we included dummies for the year of data collection. Due to the high correlation between GDP per capita and the governmental spending on health, we could not simultaneously use the two variables in our models. We opted to include the GDP per capita measure. In order to test whether the health gap between the rich and the poor is wider in countries with higher levels of inequality, we estimated a model where we introduced the interaction between Gini Index of Household Wealth and the household wealth dummies.

## Results

We first examined whether the level of wealth inequality was associated with the prevalence of anemia, the experience of child mortality and with the country's resources relevant to health (see Table 2). Because of the way our dependent variables were measured (i.e., 1 stands for the worse health status) we expected to find a positive correlation between the level of household wealth inequality and the aggregated health indicators. Our expectation was confirmed: we found that higher levels of household wealth inequality were positively correlated with higher prevalence of anemia and of experience of child mortality. Note that the correlation in the women anemia sample was half in strength in comparison with the correlations found in the child anemia and experience with child mortality samples.

**Table 2:** Ecological correlations of Gini Index of Household Wealth with average population health and other contextual measures in the three samples of LMICs.

*Correlations of Gini Index of Household Wealth with:*	Women anemia sample a	Children anemia sample b	Experience of child mortality sample c
Aggregated health	**.39**	**.76**	**.80**
Private financing of health	.07	.11	.15
Measles vaccination	**−.63**	**−.61**	**−.53**
GDP per capita	**−.72**	**−.71**	**−.63**

Note: Estimates in bold are correlation coefficients that are statistically significant for α<.05.

a : women anemia sample, dependent variable aggregated for 373735 women in 33 countries.

b : children anemia sample, dependent variable aggregated for 152485 children with age lower than 71 months in 30 countries.

c : experience of child mortality sample, dependent variable aggregated for 455692 women in 52 countries.

Furthermore, Gini Index of Household Wealth was negatively and significantly correlated with the coverage of measles vaccination - our proxy for the coverage of basic health services/effectiveness of the health sector, and with the GDP per capita. The correlation with the share of private financing of health was statistically not significant. Based on these figures, we found the first evidence that linked higher levels of inequality with worse health and with less country's resources that are relevant for population health (albeit not all).

### Inequality, resources and average health

Table 3, 4 and 5 present selected effects from multilevel models testing our hypotheses. First, we estimated a model with only an intercept allowed to vary between countries and with dummies for years (effects not presented in tables). Based on this model, we estimated the initial country level variance and the intra-class correlation coefficients (ICC). For the women anemia sample, the initial between-countries variance was.30 and the ICC was.08, for the children anemia sample, the initial between-countries variance was.75 with a corresponding ICC of.18, while for the child mortality sample, the initial between-countries variance was.52 and the ICC was.14.

**Table 3:** Results of the logistic multilevel regression for dependent variable anemia status (373735 women in 33 countries).

	Model 1	Model 2	Model 3	Model 4	Model 5
Gini Index of Household Wealth	**.25** (.09)	.12 (.09)	.10 (.08)	−.07 (.13)	.16 (.11)
Household wealth (richest 5^th^ quintile)		**−.56** (.01)	**−.44** (.02)	**−.44** (.02)	**−.29** (.09)
Household wealth (4^th^ quintile)		**−.40** (.01)	**−.31** (.01)	**−.31** (.01)	**−.19** (.07)
Household wealth (3^th^ quintile)		**−.24** (.01)	**−.18** (.01)	**−.18** (.01)	***−.10*** (.06)
Household wealth (2^nd^ quintile)		**−.12** (.01)	**−.09** (.01)	**−.09** (.01)	***−.06*** (.04)
Private financing of health				.05 (.08)	
Measles vaccination				−.13 (.10)	
GDP per capita				−.11 (.11)	
Interactions Gini Index of Household Wealth*					
Household wealth (5^th^ quintile)					.01 (.09)
Household wealth (4^th^ quintile)					−.00 (.09)
Household wealth (3^th^ quintile)					−.05 (.08)
Household wealth (2^nd^ quintile)					−.04 (.05)
*Control variables*			**Yes**	**Yes**	**Yes**
*Country level variance*	.237	.236	.214	.191	.251

Notes: Effects on the log(Y) presented with standard error of estimates in parentheses. Estimates in bold are statistically significant for α<.05, in bold + italics for α<.10. Continuous variables are standardized. All models include dummy variables for the year of data collection – effects not presented.

**Table 4:** Results of the logistic multilevel regression for dependent variable anemia status (152485 children with age less than 71 months in 30 countries).

	Model 1	Model 2	Model 3	Model 4	Model 5
Gini Index of Household Wealth	**.65** (.11)	**.47** (.11)	**.44** (.10)	**.32** (.16)	**.61** (.14)
Household wealth (richest 5^th^ quintile)		**−.73** (.02)	**−.48** (.03)	**−.48** (.03)	**−.44** (.08)
Household wealth (4^th^ quintile)		**−.41** (.02)	**−.29** (.02)	**−.29** (.02)	**−.24** (.06)
Household wealth (3^th^ quintile)		**−.22** (.02)	**−.17** (.02)	**−.17** (.02)	**−.11** (.05)
Household wealth (2^nd^ quintile)		**−.10** (.02)	**−.09** (.02)	**−.09** (.02)	**−.09** (.04)
Private financing of health				−.02 (.10)	
Measles vaccination				−.05 (.14)	
GDP per capita				−.13 (.14)	
Interactions Gini Index of Household Wealth*					
Household wealth (5^th^ quintile)					**−.19** (.10)
Household wealth (4^th^ quintile)					***−.18*** (.09)
Household wealth (3^th^ quintile)					−.12 (.08)
Household wealth (2^nd^ quintile)					−.02 (.07)
*Control variables*			**Yes**	**Yes**	**Yes**
*Country level variance*	.343	.352	.275	.266	.364

Notes: Effects on the log(Y) presented with standard error of estimates in parentheses. Estimates in bold are statistically significant for α<.05, in bold + italics for α<.10. Continuous variables are standardized. All models include dummy variables for the year of data collection – effects not presented.

**Table 5:** Logistic multilevel regression estimates for dependent variable experience of child mortality (455692 women in 52 countries).

	Model 1	Model 2	Model 3	Model 4	Model 5
Gini Index of Household Wealth	**.61** (.06)	**.35** (.05)	**.38** (.05)	**.22** (.08)	**.21** (.08)
Household wealth (richest 5^th^ quintile)		**−1.09** (.03)	**−.17** (.04)	**−.17** (.04)	**−.17** (.07)
Household wealth (4^th^ quintile)		**−.77** (.02)	**−.13** (.03)	**−.13** (.03)	−.08 (.05)
Household wealth (3^th^ quintile)		**−.42** (.02)	−.02 (.02)	−.02 (.02)	.00 (.05)
Household wealth (2^nd^ quintile)		**−.13** (.01)	**.07** (.01)	**.07** (.01)	.04 (.03)
Private financing of health				.02 (.04)	
Measles vaccination				**−.21** (.06)	
GDP per capita				−.07 (.06)	
Interactions Gini Index of Household Wealth*					
Household wealth (5^th^ quintile)					.02 (.07)
Household wealth (4^th^ quintile)					.09 (.06)
Household wealth (3^th^ quintile)					.04 (.06)
Household wealth (2^nd^ quintile)					.04 (.04)
*Control variables*			**Yes**	**Yes**	**Yes**
*Country level variance*	.145	.137	.110	.083	.062

Notes: Effects on the log(Y) presented with standard error of estimates in parentheses. Estimates in bold are statistically significant for α = .05. Continuous variables are standardized. Models also include dummy variables for the year of data collection – effects not presented.

In Model 1 (Table 3, Table 4 and Table 5), we estimated the uncontrolled effect of Gini Index of Household Wealth. As expected, we found that higher Gini Index of Household Wealth was significantly related with higher chance to be tested with anemia or to the chance of women to experience the death of a child. The effect was more than double in the children anemia sample in comparison to the effect in the women anemia sample (.65 compared to.25). Additionally, around 70% of the country-level variance for the child mortality sample and around 54% for the children anemia sample could be attributed to differences in household wealth inequality between the countries, but only around 10% for the women anemia sample.

In Model 2 (Table 3, Table 4 and Table 5), we added the household wealth and we found that the odds to be tested with anemia of the women living in one of the richest 20% households in the sample were.57 lower compared to the women living in one of the poorest 20% households (exp(-.56)). Similarly, the odds of the richest to the poorest 20% women to experience the death of a child was.33 lower (exp(-1.09)). Thus, the health gap between the richest to the poorest women is larger when it comes to child mortality experiences than in the case of anemia. With regard to the children sample, the odds of the richest to the poorest 20% children to be tested with anemia was.48 lower (exp(-.73)).

In addition, the inclusion of household wealth in the model led to the reduction of the effect of Gini Index of Household Wealth of around 52%, 28% and 43% in the women anemia sample, children anemia sample and experience of child mortality sample. However, this effect continued to be statistically significant for the child anemia sample and for the experience of child mortality sample. Thus, our results provided support for the expectation that the empirical relationship between inequality and health is, at least partially, a result of a compositional effect due to the distribution of material resources in the population. This conclusion did not change when adding the additional individual level control variables (Model 3 in Table 3, Table 4 and Table 5).

In Model 4 (Table 3, Table 4 and Table 5), we added the country's resources relevant to health. We first observed that the effect of Gini Index of Household Wealth was further reduced, but for the child mortality sample and for the children anemia sample it remained statistically significant. However, the country's resources relevant to health had weak and mostly not significant relationships with the average health in the three samples. Still, compared to Model 3, the account of these contextual measures decreased the country-level variance with around 11% for the women anemia sample, around 3% for the children anemia sample and with around 24% for the child mortality sample. Thus, based on the above, we found some support for hypothesis 3.

### Inequality and the health gap

In Model 5 in Table 3, Table 4 and Table 5, we added the interactions between Gini Index of Household Wealth and the quintiles of household wealth contrasting the respondents living in absolute poverty (the 20% poorest in our samples) to the rest. The interactions were statistically not significant for p<.05 for both our dependent variables and in all the three samples. However, the interaction coefficients found in the children anemia sample were all negative and for the richest 40% of households they were stronger and were statistically significant for p<.06. Based on these results, we did not find conclusive evidence that countries with higher levels of wealth inequality had higher levels of health inequality compared to countries with lower levels of wealth inequality.

### The role of sample composition

**Figure 2:**
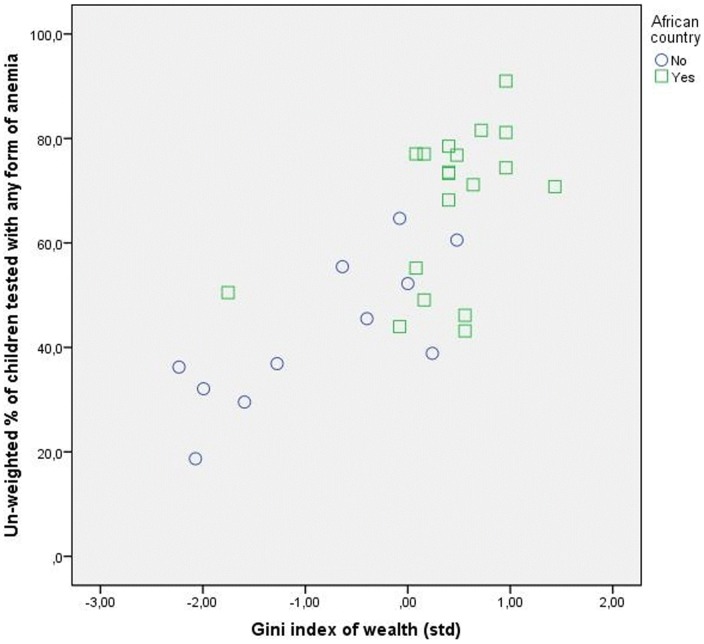
Household wealth inequality and the anemia status of children in African and non-African countries.

**Figure 3:**
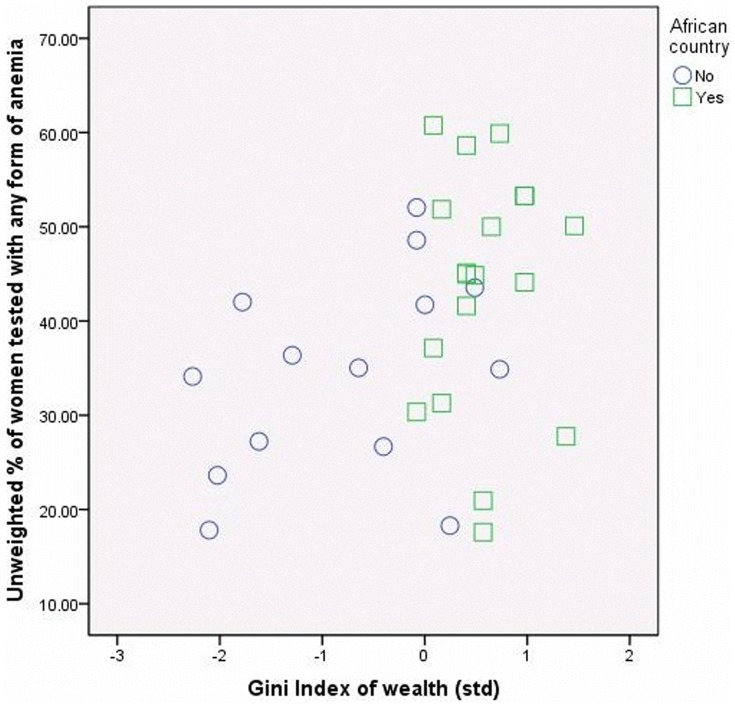
Household wealth inequality and the anemia status of women in African and non-African countries.

**Figure 4:**
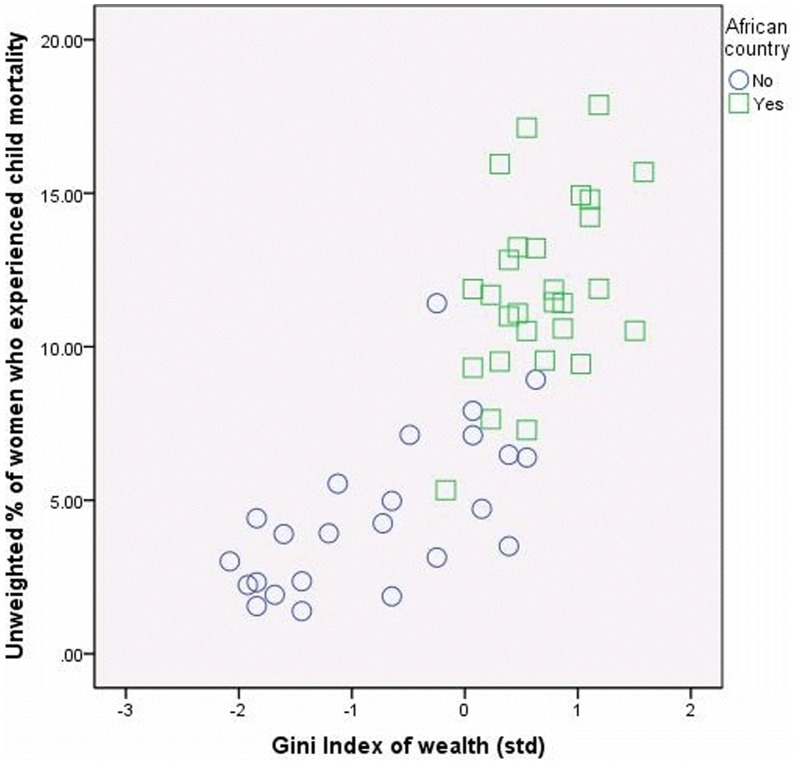
Household wealth inequality and the experience of child mortality of women in African and non-African countries.

By inspecting the scatter plots of the Gini Index of Household Wealth with the aggregated dependent variables we observed a clear clustering of African/non-African countries (See Figs. 2–4). This observation raised concerns regarding the role of the sample composition for our conclusions. We briefly explored this possibility for the child mortality sample. Unfortunately the number of countries in the women anemia sample and children anemia samples were too low and we could not perform separate detailed analyses.

We re-estimated Model 4 in Table 5 by including a dummy variable for the non-African countries and we observed a decrease of the effect of Gini Index of Household Wealth to the point where it turned statistically not-significant. Analyses on separate sub-samples showed that the particularity of the African countries is a feature of their socio-economic profile. For the sample of 28 African countries the uncontrolled significant effect of Gini Index of Household Wealth was fully explained when we took into account the individual level variables. Furthermore, the only contextual characteristic that decreased the chance of experiencing child mortality was the measles vaccination coverage, i.e., the measure of coverage of basic health services/effectiveness of health system. In the subsample of 24 non-African countries the uncontrolled significant effect of Gini Index of Household Wealth was explained both by compositional effects of individual material resources and by the country's characteristics relevant to health. In addition, in the non-African countries, both the measles vaccination coverage and the share of private financing were significantly related to the chance of women to experience child mortality.

## Conclusions and Discussion

In the present study, we set out to investigate the relationship between inequality and two measures of health: anemia status of women and their children and the experience of child mortality. We extended the previous literature by looking at these relationships among LMICs, by using health information on individuals collected in as much as 52 countries, and by analyzing the relationship between inequality with average health and health inequality. We first examined whether countries with higher levels of inequality displayed worse health, and whether this relationship was robust to population composition. Second, we explored a potential mediation path linking inequality to health via the countries' resources relevant to health. Third, we were interested to see if the health gap between the rich and the poor was wider in more unequal LMICs. Based on our analyses we derive the following conclusions.

First, we found evidence supporting the idea that higher wealth inequality was associated with worse health in the LMICs. In our models, the chance of women and children to be tested with a form of anemia and the chance of women to experience child mortality was significantly higher with higher wealth inequality. This conclusion is in line with findings from other studies in the LMICs, albeit on different health outcomes or preconditions for health. To cite only a few results, wealth inequality was found to be positively associated with the chance to be tested HIV seropositive in sub-Saharan Africa [47] or inequality in income was found to be associated with higher levels of pre-term birth [51] and with child malnutrition [52].

Second, our results point toward the non-linear relationship between health and material resources within countries as the main reason why we initially found a significant association between higher household wealth inequality and worse health. In more unequal countries there are more people that have precarious living conditions and very low levels of material resources compared to countries where the material resources are more evenly distributed. Since in the LMICs large part of the health funding is based on the out-of-pocket payments and the policies to support the poor are scarce, material resources are very important for health and strong differences in individual wealth translate into strong differences in health. Thus, in the LMICs the relationship between inequality and health is, to a large extent, a “statistical artefact”.

However, composition effects had different weight for explaining the relationship between household wealth inequality and health, depending on the countries in the sample. We found that African countries had a specific profile – these are the countries where the poorest individuals are concentrated, and this concentration of low material resources at individual level fully explained the relationship between inequality and health. In comparison, among the non-African countries the health system characteristics mattered more for explaining the observed relationship between inequality and health. It is possible that the countries from the two geographic areas systematically differ in the development and accessibility of health services or in the type of public policies and welfare arrangements aimed to protect the poor. For example, a good territorial coverage of health infrastructure will benefit a larger segment of the population, especially the poor population residing in remote rural areas. Also, policies that permit poor individuals to access the health services and infrastructure will increase the use of medical care, with positive effects on the overall health of the population. Due to data limitation we could not pursue these alternative explanations but we encourage future research to systematically examine the role of welfare arrangements and policies targeting the population's access to health infrastructure and services in relation to health.

Third, we found evidence suggesting that inequality could have a genuine contextual effect on health in the LMICs. Even after accounting for the differences between individuals in terms of household wealth and other characteristics, in those LMICs with higher household wealth inequality more women experienced child mortality and more children were tested with anemia. We examined one potential mechanism that could be behind this contextual effect: the argument that in countries with higher inequality, the country's resources relevant to health are less developed. Our results provided some support for this proposition: around 40% from the effect of Gini Index of Household Wealth on the propensity to experience child mortality was explained when the country's resources relevant to health were taken into account. Similarly, the reduction was around 48% in the children anemia sample. In the light of the robust and convincing economic literature linking inequality to reduced growth and lower investment in public goods and human capital [27], [28], [30], it is reasonable to accept these results as a tentative empirical support for the proposed mechanism. However, because our data was cross-sectional, no strong claims regarding a causal chain can be made.

Fourth, we found that the relationships between the characteristics of the health system and our health measures were very weak and in majority of cases statistically not significant. In addition, we found evidence suggesting that health institutions had different weight for health among the African and non-African countries and for the two health indicators. These findings suggest that the mediating effect of health institutions could differ between different samples of countries and could be outcome specific. In this particular study, we cannot assert which of these possibilities has more weight. An answer to this issue would shed more light in the functioning of health institutions for health in the LMICs, with important implications for public policies.

Finally, our results indicate that a very prominent policy idea, i.e., that higher inequality is associated with a wider health gap between rich and poor, is not supported by empirical evidence in an indisputable manner. It is not clear to us the reason why the health gap in anemia and the experience of child mortality between rich and poor women was not lower in those LMICs with lower household wealth inequality. Our results could be due to the choice of health measures, inequality measure, or maybe to some other factors that we did not account for. This remains an open question for future research.

We note that we only took into account two measures of physical health in our analyses, i.e., anemia status and the experience of child mortality, and we only looked at two potential explanations for the empirical relationship between inequality and health. However, inequality might relate to health via alternative mechanisms, e.g., inequality could act like a contextual stressor [5] or it could reduce trust and social cohesion [53] and via these pathways it could lead to health problems. In addition, our investigated population is restricted only to women and infants, and thus we cannot be certain if our results also hold for the male population. However, since the Millennium Development Goals [15] has a special focus on the health of women and children, the lack of generalizability of our conclusions to the whole population does not diminish their importance.

While the above are limitations of our research, we note that the measures that we utilized are (mostly) objective health measures, collected either via a blood test or via birth histories. Thus, the chance to be biased by the subjects' knowledge or willingness to declare the truth is null or extremely low. By using these two measures of physical health, we addressed concerns regarding the equivalence of health measures in cross-national comparative research [54], [55] and increase the validity of our findings.

To sum up, our contribution shows that, in general, reducing inequality in the LMICs might not result in better average health of women and children. Instead, a more effective approach is to improve the wealth *among the poor households*, which will result in better living conditions, better nutrition and more resources for accessing medical services and, as a result, overall better health. Targeted policies aimed at improving literacy, developing community infrastructure and increasing the connections between rural and urban areas have the long term potential of sustainable improvement of the wealth of the poor in the LMICs. Of course, alleviating poverty among the poor will also lead to the reduction of overall inequality with potential spillover effects on economic growth and investments in public health infrastructure and services, which in turn could also have a positive impact on health.

## Supporting Information

S1 File **S1 File Covers the structure of the samples used in the analyses, i.e., the countries, the waves and the years of data collection.**
(XLSX)
